# Advances in Non-Pharmacological Strategies for DOMS: A Scoping and Critical Review of Recent Evidence

**DOI:** 10.3390/jfmk10040452

**Published:** 2025-11-20

**Authors:** Luigi Di Lorenzo, Alfonso Maria Forte, Valeria Agosti, Francesco Forte, Tiziana Lanciano, Nicola Pirraglia, Carmine D’Avanzo

**Affiliations:** 1 Rehabilitation, Department Life and Health Professions, Link Campus University, 00165 Roma, Italy; 2 Istituto Neurologico Mediterraneo, 86077 Pozzilli, Italy; 3 Biomedical Research Center, Gruppo Forte, 84124 Salerno, Italy; 4 Department of Humanities, Philosophy and Education, University of Salerno, 84084 Fisciano, Italy

**Keywords:** DOMS, muscle soreness, cryotherapy, massage, vibration therapy, sports recovery, fascial pain, rehabilitation, athletes

## Abstract

**Background**: Delayed Onset Muscle Soreness (DOMS) is a transient, exercise-induced condition characterized by muscle pain, stiffness, and functional impairment, particularly following eccentric or high-intensity physical activity. Recent advances in diagnostic imaging, neurophysiology, and therapeutic techniques have led to a reassessment of DOMS pathophysiology and management. **Objective**: This scoping review aims to critically evaluate non-pharmacological strategies for DOMS management, focusing on clinical studies published between 2020 and 2025. Emphasis is placed on physical, thermal, neurophysiological, and nutritional interventions in athletic populations. **Methods**: A comprehensive literature search was conducted using PubMed, Scopus, and Web of Science. Included studies were randomized controlled trials, systematic reviews, meta-analyses, and high-quality scoping reviews. Methodological quality was assessed using PEDro, AMSTAR 2, and ROBIS tools. Key outcome measures included pain (VAS), functional recovery (ROM, performance), biochemical markers (CK, IL-6), and neuromuscular activation (iEMG). **Results**: Twenty-five studies met the inclusion criteria. Emerging strategies such as cryosauna, vibration therapy, percussive massage, and polyphenol supplementation demonstrated significant benefits in reducing DOMS-related symptoms and enhancing recovery. Evidence supports the integration of multimodal, personalized interventions over monotherapies. Imaging techniques (7T MRI, ultrasound) confirmed microstructural muscle changes consistent with DOMS, strengthening diagnostic precision. **Conclusions**: Non-pharmacological approaches to DOMS have evolved considerably, highlighting the importance of combining mechanical, thermal, and nutritional modalities. Personalized, multimodal recovery strategies appear most effective for symptom relief and performance restoration. Future studies should aim to standardize treatment protocols and outcome measures to improve clinical applicability.

## 1. Introduction

Delayed Onset Muscle Soreness (DOMS) often shows up after intense or unfamiliar workouts, leaving muscles feeling stiff and achy. This natural response to physical exertion can make everyday movements more challenging and temporarily impact athletic performance or training consistency. DOMS has long been linked to eccentric muscle movements, but recent studies have shed light on its more intricate causes. This deeper understanding has led to a broader array of treatment approaches [1,2]. Historically, six main theories explained DOMS, including concepts like lactic acid accumulation, muscle spasms, and inflammation, among others [2]; however, recent evidence emphasizes the critical role of muscle micro-tears, inflammatory responses, and connective tissue damage as central to DOMS development [1,3].

Kancherla [1] highlights that micro-injuries at the myofibrillar level trigger a cascade of inflammatory processes, leading to the pain and stiffness characteristic of DOMS. Among the early theories, the role of lactic acid has long been considered obsolete. Current evidence supports a multifactorial pathophysiology involving myofibrillar microdamage, inflammatory cascades, and oxidative stress. These include the release of proinflammatory cytokines and oxidative stress, which play a significant role in muscle soreness and reduced functionality [1]. While rest, physical therapy, and NSAIDs have traditionally been the go-to treatments for managing Delayed Onset Muscle Soreness (DOMS), recent advancements in holistic therapies and innovative physical techniques are proving highly effective, particularly for professional athletes dealing with acute and sub-acute myalgia.

Recognizing the potential of these emerging approaches, our research and clinical focus have shifted toward evaluating their efficacy in real-world applications. This scoping review aims to explore and highlight the latest minimally invasive treatments for acute, sub-acute, and chronic muscle soreness in athletes. Consistent with a scoping review framework, this work also aims to systematically map the breadth of non-pharmacological interventions studied between 2020 and 2025, identify thematic clusters within the literature, and highlight existing gaps that warrant further investigation through high-quality randomized controlled trials (RCTs).

Our goal is to assess the current application, effectiveness, and broader potential of these methods in addressing this common issue within the sports community. This review aims to critically assess current evidence on the efficacy of emerging non-pharmacological interventions for DOMS, with a focus on studies published between 2020 and 2025.

Given the high prevalence of DOMS among both recreational and professional athletes, and the increasing demand for safe, non-pharmacological recovery strategies, a systematic synthesis of recent evidence is essential to inform clinical decision-making and optimize performance outcomes

## 2. Materials and Methods

We conducted a SCOPING review of literature using PubMed, Scopus, and Web of Science. Studies included were RCTs, systematic reviews, and meta-analyses from 2020 to 2025, focused on DOMS management in athletes. This work is structured as a scoping review, with critical synthesis of findings, following the framework of Arksey and O’Malley and the PRISMA-ScR guidelines. Only English-language human studies were considered. Excluded were animal studies, single case reports, and interventions based solely on pharmacologic therapy. Consistent with the scoping review methodology, all high-quality sources of evidence—including RCTs, systematic reviews, meta-analyses, and scoping reviews—were included to provide a comprehensive mapping of current knowledge. This inclusive approach allows the identification of research trends, methodological gaps, and evidence redundancy, which would not be possible by restricting inclusion to primary studies alone.

**Figure 1:**
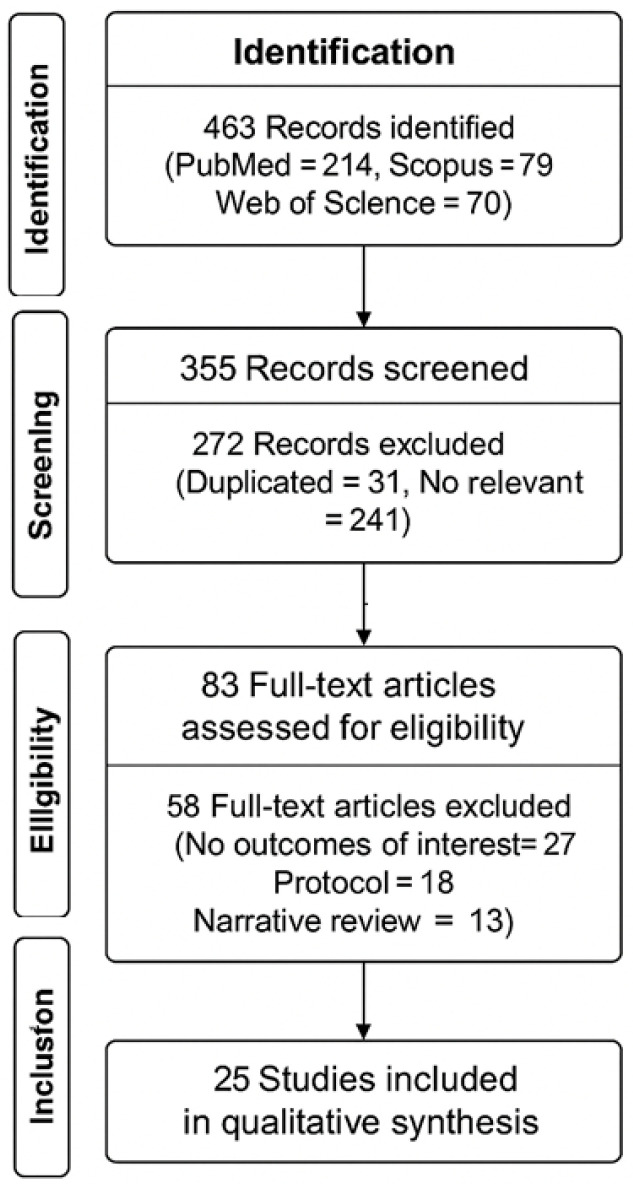
Studies screened.

To ensure comprehensiveness, reference lists of relevant studies were also reviewed (Figure 1). Investigators independently conducted the search and selection process. Titles and abstracts were screened initially, followed by a full-text assessment for eligible studies. Ethical approval and patient consent were not required, as the review relied on previously published research without involving patient interactions or altering care. Discrepancies during the selection process were resolved by the first author (LDL) through consensus.

In accordance with the methodological framework of scoping reviews proposed by Arksey and O’Malley and expanded by the PRISMA-ScR guidelines [3,4], this study was designed to systematically map the available evidence on non-pharmacological strategies for DOMS in athletes.

Data were charted thematically according to five main intervention domains identified across the included studies: (1) physical and mechanical therapies (e.g., foam rolling, vibration), (2) thermal interventions (e.g., cryosauna, hydrotherapy), (3) nutritional strategies (e.g., BCAA, polyphenols), (4) neurophysiological techniques (e.g., electrical stimulation, PNF), and (5) diagnostic imaging and biomarkers.

This thematic mapping enabled us to summarize the breadth of current clinical evidence, identify well-supported versus under-researched interventions, and highlight gaps that warrant further investigation through high-quality randomized controlled trials (RCTs). To evaluate the effectiveness of various non-pharmacological approaches, the retrieved studies were analyzed in thematic categories, with particular emphasis on recovery-related outcomes such as pain reduction (VAS), functional performance (e.g., vertical jump, torque), biochemical markers (CK, IL-6), and neuromuscular activation (iEMG).

Finally, this scoping review did not have a registered protocol; however, it was conducted in accordance with the PRISMA-ScR (Preferred Reporting Items for Systematic Reviews and Meta-Analyses extension for Scoping Reviews) guidelines, ensuring methodological transparency and reporting rigor.

## 3. Results

The literature search yielded approximately 23 full-text articles identified as relevant and subsequently screened for inclusion, some of those published between 2020 and 2025, that met the inclusion criteria (Table 1). This scoping review included randomized controlled trials (RCTs), systematic reviews, meta-analyses, updated clinical recommendations, and high-quality scoping reviews. The evidence base primarily addresses physical, neurophysiological, and integrative modalities for managing Delayed Onset Muscle Soreness (DOMS).

Clinically, DOMS is characterized by localized muscle tenderness, stiffness, and pain, typically arising 24–72 h following eccentric or high-intensity exercise. These symptoms are often confused with acute muscle strain, although imaging and functional recovery profiles help distinguish DOMS as a transient, self-limiting inflammatory response. DOMS commonly follows unaccustomed or high-load eccentric activity and is associated with temporary declines in strength, range of motion (ROM), and performance.

The following section summarizes the most recent and relevant findings from clinical trials and systematic evidence, focusing above all on those published between 2020 and 2025. A structured comparative summary of the most relevant clinical trials and reviews published between 2020 and 2025 is presented in Table 2, providing an overview of study design, interventions, outcomes, and conclusions.

To further evaluate the scientific rigor of the selected literature, we conducted a qualitative methodological assessment using appropriate standardized tools: the PEDro scale for randomized controlled trials (RCTs), AMSTAR 2 for scoping and systematic reviews, and ROBIS for meta-analyses. Table 3 summarizes this assessment, categorizing the studies by type and reporting the assigned quality score or bias level, along with a brief qualitative evaluation. Most included RCTs demonstrated moderate to high methodological quality, while the reviews and meta-analyses showed varied but generally acceptable standards of evidence synthesis [5,6,7,8,9,10,11,12,13,14,15,16,17,18,19,21,22,23,24,25,26,27,28,29,30,31,32].

## 4. Discussion

Historically, DOMS was attributed to lactic acid accumulation; however, this theory has been refuted. Current evidence highlights microtrauma, inflammation, and nociceptor sensitization as primary mechanisms. In recent years, a growing body of research has emerged, featuring significant trials assessing the effectiveness of innovative physical therapies and thermotherapies for the management of Delayed Onset Muscle Soreness (DOMS). Current pathophysiological understanding emphasizes that DOMS is not primarily due to lactic acid accumulation, but results from eccentric muscle fiber disruption, perimysial connective tissue damage, and sensitization of nociceptors—particularly in the fascia [12]. The condition is commonly associated with inflammation, elevated serum creatine kinase (CK), leukocytosis, and acute-phase reactants, which typically peak within 1–7 days after intense or unfamiliar exercise.

DOMS can also impair glycogen resynthesis, particularly in endurance athletes, potentially delaying full functional recovery [25]. For this reason, its management requires a multifactorial strategy that includes diagnostic accuracy, therapeutic interventions, and prevention. From a diagnostic standpoint, MRI has become an essential tool for identifying muscle edema and structural changes in DOMS, offering superior sensitivity compared to ultrasound. Notably, the recent application of 7-Tesla MRI [5] has enabled the early detection of microstructural muscle damage, representing a significant diagnostic breakthrough. Nevertheless, ultrasound remains a valuable tool in sports settings due to its accessibility, revealing characteristic findings such as diffuse echogenicity, muscle thickening, and fascial swelling [6].

At the cellular level, DOMS involves sarcomere disruption, cytoskeletal breakdown, and a secondary inflammatory cascade with neutrophil and macrophage infiltration. Satellite cells contribute to regeneration in the subacute phase, yet nociceptor sensitization may prolong pain beyond structural healing [7,8,10,12].

In a randomized controlled study, Li and colleagues (2025) [26] demonstrated that applying percussive massage therapy for 40 min led to notable improvements in pain levels—as measured by the Visual Analog Scale (VAS)—as well as enhancements in neuromuscular activation (iEMG), range of motion (ROM), and functional performance (e.g., vertical jump). These benefits were more pronounced than those observed with static stretching or shorter Percussive Massage Therapy (PMT) durations, suggesting a dose–response relationship for this intervention [26].

Similarly, Cheng et al. (2025) [27] provided evidence that an integrated protocol involving vibration training in combination with kinesio taping yielded superior results compared to each modality in isolation. Specifically, the combined intervention led to greater reductions in perceived pain, lower levels of inflammatory biomarkers such as interleukin-6 (IL-6) and creatine kinase (CK), and improved muscular torque generation. This highlights the potential of synergistic, multimodal strategies in optimizing recovery from DOMS [27].

Wolska and collaborators (2023) [9] confirmed the clinical efficacy of whole-body cryosauna when administered between 48 and 72 h after intense physical activity. Their findings showed significant reductions in muscle stiffness, leukocyte count, and creatine kinase levels, reinforcing the role of delayed cryotherapy as a systemic anti-inflammatory and recovery-enhancing intervention [9].

The phenomenon of exercise-induced analgesia, which occurs during low-intensity aerobic or neuromuscular activity, has been repeatedly observed to attenuate DOMS-related discomfort. This supports the use of structured active recovery protocols as a clinically viable alternative to complete rest, potentially preserving mobility while reducing pain perception [11].

When comparing recovery strategies, studies have found that Proprioceptive Neuromuscular Facilitation (PNF) combined with localized ice massage demonstrates significantly greater reductions in both muscle soreness and stiffness compared to static stretching. This suggests that interventions involving neuromuscular engagement and targeted thermotherapy may offer added value for post-exercise recovery [28].

Supporting these findings, the umbrella review conducted by Wiecha et al. (2024) [11] concluded that traditional stretching protocols—often included in recovery routines—do not offer meaningful clinical benefits in the management of DOMS. Based on a synthesis of available evidence, the authors recommend de-emphasizing static stretching as a primary recovery strategy [11].

Foam rolling has consistently been shown to improve tissue tone, flexibility, and elasticity, as reported by Szajkowski et al. (2025) [29]. Namely, foam rolling has consistently been shown to improve tissue tone, flexibility, and elasticity. While some studies report pain-reducing effects, others find limited or inconsistent impact on perceived soreness, indicating a primarily mechanical role. The addition of vibration to foam rolling—commonly referred to as VFR—has been associated with improved outcomes, particularly in joint range of motion and perceived fatigue. However, these benefits must be interpreted cautiously due to limitations in sample sizes and methodological quality. However, while some studies report pain-reducing effects, others find limited or inconsistent impact on perceived muscle soreness, indicating that its role may be more mechanical than analgesic in nature [29].

The addition of vibration to foam rolling—commonly referred to as VFR—has been associated with improved outcomes, particularly in terms of increasing joint range of motion and reducing perceived fatigue. Nonetheless, these benefits must be interpreted cautiously, as the supporting studies often suffer from limitations in sample size, follow-up duration, and methodological quality [30].

Acupuncture has emerged as a viable non-pharmacological option for modulating pain perception and reducing muscular stiffness in the context of DOMS. Despite these benefits, current evidence suggests it does not significantly enhance muscle force production or athletic performance, thereby limiting its utility in performance-focused recovery strategies [31].

In contrast, the use of electrical stimulation as a recovery modality, as evaluated by Gussoni et al. (2023) [16], failed to produce statistically significant improvements in DOMS-related outcomes compared to placebo conditions. These findings suggest that, in its current form, electrical stimulation may not be effective as a standalone intervention [16].

From a nutritional standpoint, both branched-chain amino acids (BCAAs) [22] and polyphenol-rich supplements such as TensLess^®^ [18] have shown promising results in mitigating the symptoms of DOMS and facilitating recovery processes. However, these results are primarily drawn from small-scale or preliminary studies, and the evidence base would benefit from large, multicenter randomized controlled trials to confirm efficacy and determine optimal dosing protocols.

Hydrotherapy continues to play a foundational role in post-exercise recovery programs. Among its modalities, cold water immersion has shown consistent benefit in reducing subjective soreness. In contrast, contrast baths—which alternate between hot and cold temperatures—do not appear to offer additional therapeutic advantage. Cryosauna, particularly at −110 °C and when applied 48 to 72 h after training, has demonstrated efficacy in lowering muscle stiffness and reducing circulating markers of muscle damage such as CK and leukocytes [9].

Whole-body vibration (WBV) has been increasingly studied as a means to enhance recovery in elite athletic populations. Some studies report reductions in muscle soreness and improvements in neuromuscular efficiency following WBV protocols [14,17]. Nevertheless, the literature remains mixed, with certain trials—such as that of Scudamore et al.—showing functional performance improvements without corresponding reductions in pain perception [19]. Similarly, Wahl et al. reported no discernible benefit of aqua cycling over passive rest, underscoring the variability in outcomes among different recovery modalities [23].

The role of fascial tissue in DOMS pathophysiology is gaining increasing attention, prompting the exploration of targeted interventions such as vibration-based therapies, manual myofascial release, and nutraceutical strategies aimed at enhancing fascial health. Concurrently, evidence also points to the involvement of the sympathetic nervous system in modulating both pain perception and physiological recovery, particularly in high-level athletes who face frequent and intense loading cycles [13].

Compression garments and thermotherapeutic techniques have been endorsed by Schroeter et al. (2024) [24] as valuable tools not only in the treatment but also in the prevention of DOMS. Their analysis highlights that early application of these modalities may mitigate symptom onset and improve recovery trajectories [24].

A comprehensive meta-analysis by Chen et al. (2024) [15] supports the use of combination protocols—such as integrating cryotherapy with massage and targeted supplementation—as the most effective and versatile approach for DOMS management. These multimodal strategies appear to offer additive or synergistic benefits, particularly when tailored to the specific recovery needs of athletic and clinical populations [15].

It is also important to acknowledge that certain forms of training themselves can exacerbate DOMS, especially when involving multiple high-impact or novel stimuli. Doma et al. (2021) reported that a single multimodal plyometric training session resulted in measurable decrements in neuromuscular performance and elevated soreness levels lasting up to 48 h post-exercise, underscoring the need to balance training intensity with recovery planning [21].

Despite these advances, important gaps remain in the literature. Indeed, many studies are limited by small sample sizes, short-term follow-up, and heterogeneity in outcome measures, which hinders data pooling and generalizability. Moreover, there is a lack of standardized treatment protocols and limited evidence on population-specific responses, such as in youth or female athletes and individuals with recurrent myofascial pain. In addition, few investigations have addressed long-term outcomes, return-to-performance timelines, or the integration of objective neuromuscular biomarkers into clinical decision-making. Conversely, some interventions, such as electrical stimulation or aqua cycling, have shown limited efficacy in clinical trials and therefore may not be suitable as primary recovery strategies.

Future research should focus on large-scale, well-powered randomized controlled trials that employ harmonized outcome measures and stratify results by athletic level and sex. Furthermore, mechanistic studies integrating imaging, neurophysiological, and biochemical analyses are needed to enhance our understanding of optimal intervention timing and potential synergistic effects.

This review has several limitations. As a scoping review, it does not provide a quantitative synthesis (e.g., meta-analysis), and no formal risk-of-bias tool was applied to all included studies. Although we evaluated the methodological quality of RCTs and reviews using appropriate tools (PEDro, AMSTAR 2, ROBIS), the absence of a formal meta-analysis and protocol registration is a limitation. Moreover, intervention heterogeneity and lack of uniform outcome measures reduce cross-study comparability.

## 5. Conclusions

Non-pharmacological approaches to DOMS, including cryotherapy, vibration therapy, and polyphenol supplementation, have shown promising results in reducing muscle soreness and enhancing functional recovery. Evidence supports the superiority of multimodal, personalized protocols over isolated treatments.

### Practical Recommendation

Based on current findings, the most effective DOMS management protocol includes delayed cryosauna (48–72 h post-exercise), percussive massage (30–40 min), and polyphenol supplementation. Foam rolling and active recovery can support outcomes, while static stretching and electrical stimulation show limited benefit and are not recommended as primary strategies.

The effective management of Delayed Onset Muscle Soreness (DOMS) requires a comprehensive, evidence-based approach tailored to the physiological and performance needs of each athlete. Current strategies—such as cryotherapy, structured active recovery, and nutritional supplementation—offer symptomatic relief and functional support, though their effectiveness remains context-dependent.

Emerging modalities like cryosauna have shown encouraging results in reducing muscle stiffness, inflammatory markers, and recovery time due to their intense whole-body cooling effects.

Similarly, acupuncture has been revisited as a neuromodulatory tool, capable of reducing pain perception and enhancing muscle activation, although its impact on force production and performance restoration remains uncertain.

Sport-specific protocols for DOMS should aim to integrate these therapies into individualized regimens, emphasizing multimodal interventions that act at both systemic and local levels. While the underlying molecular mechanisms of DOMS are increasingly understood—including fascial involvement, sympathetic regulation, and microstructural tissue changes—therapeutic priorities should remain oriented toward rapid symptom control, functional restoration, and injury prevention.

Despite the recent progress in diagnostic imaging and therapeutic innovation, DOMS continues to represent a complex and multifaceted challenge at the crossroads of sports medicine, rehabilitation, and human performance optimization.

Future research should focus on standardizing treatment protocols, identifying athlete-specific response patterns, and leveraging novel biomarkers to guide personalized recovery strategies.

## Figures and Tables

**Table 1:** Flow Diagram Table—Literature Selection Process (2020–2025).

Stage	Description	Number of Records
Identification	Records identified through database searching (PubMed, Scopus, Web of Science)	461
Screening	Records after duplicates removed	355
	Records screened by title and abstract	355
	Records excluded (not related to DOMS or not meeting inclusion criteria)	272
Eligibility	Full-text articles assessed for eligibility	83
	Full-text articles excluded (e.g., protocol only, pharmacological-only, incomplete outcomes)	58
Included	Studies included in qualitative synthesis	23

**Table 2:** Evidence Matrix of Included Studies on Non-Pharmacological Strategies for DOMS (2020–2025).

Author (Year)	Journal	Study Type	Intervention/Focus	Key Findings/Contribution	Thematic Category
Kancherla A (2023) [1]	*Ann Innov Med*	Management Update	Overview of DOMS management	Summarizes recent trends and therapeutic directions	General Overview
Sonkodi B et al. (2022) [2]	*J Funct Morphol Kinesiol*	Research	Neuronal microdamage & reflex delay	Highlights sensory neuron injury in DOMS	Neurophysiology
Heiss R et al. (2024) [5]	*Ann Anat*	Imaging Study	7T MRI for early detection	Demonstrates advanced imaging sensitivity to microdamage	Imaging/Biomarkers
Longo V et al. (2016) [6]	*J Ultrasound Med*	Imaging Study	Ultrasound markers	Shows fascial thickening and echogenicity in DOMS	Imaging/Biomarkers
Hotfiel T et al. (2018) [7]	*Sportverletz Sportschaden*	Review	Pathogenesis & diagnostics	Reviews DOMS etiology, structural markers	Pathophysiology
Xue X et al. (2023) [8]	*BMC Musculoskelet Disord*	RCT	Kinesio taping + compression sleeves	Reduces pain and improves recovery markers	Mechanical Therapy
Wolska B et al. (2023) [9]	*Front Physiol*	RCT	Cryosauna (−110 °C)	Reduces muscle damage markers and stiffness	Thermal Therapy
Mizumura K, Taguchi T (2024) [10]	*J Physiol Sci*	Review	Neurochemical mechanisms	Explores pain mediators and DOMS-related discomfort	Neurophysiology
Wiecha S et al. (2024) [11]	*J Clin Med*	Umbrella Review Protocol	Physical therapy strategies	Maps physical modalities for DOMS management	Mechanical Therapy
Wilke J, Behringer M (2021) [12]	*Int J Mol Sci*	Review	Fascia involvement	Reframes DOMS pathogenesis from a fascial perspective	Fascial/Connective Tissue
Fleckenstein J et al. (2021) [13]	*Front Physiol*	RCT	Sympathetic system role	Shows autonomic modulation of DOMS symptoms	Neurophysiology
Akehurst H et al. (2021) [14]	*J Orthop Surg Res*	RCT	Whole-body vibration	Improves soreness and neuromuscular performance	Mechanical Therapy
Chen R et al. (2024) [15]	*BMC Musculoskelet Disord*	Meta-analysis	Cryotherapy & hydrotherapy	Confirms benefit of cryo/hydrotherapy in DOMS	Thermal Therapy
Hotfiel T et al. (2018) [7]	*Sportverletz Sportschaden*	Review	Prevention & treatment of DOMS	Updates recommendations on intervention timing	General Overview
Gussoni M et al. (2023) [16]	*J Funct Morphol Kinesiol*	RCT	Electrical stimulation	No significant effects vs. placebo	Neurostimulation
Iodice P et al. (2019) [17]	*Eur J Appl Physiol*	Therapy Study	High-frequency vibration	Reduces pain and posture alterations	Mechanical Therapy
Romain C et al. (2017) [18]	*Phytother Res*	Nutritional Study	TensLess^®^ (polyphenols)	Reduces soreness, improves recovery	Nutritional Therapy
Scudamore EM et al. (2021) [19]	*J Exerc Sci Fit*	Performance Study	Foam rolling	Improves task performance, unclear pain effect	Mechanical Therapy
Farias-Junior LF et al. (2019) [20]	*Physiol Behav*	Comparative Study	HIIE vs. MICE exercise effects	Similar DOMS outcomes across modalities	Exercise Modality
Doma K et al. (2021) [21]	*J Sports Med Phys Fitness*	Acute Effects Study	Plyometric training	Increases DOMS, impairs neuromuscular performance	Exercise Modality
Weber MG et al. (2021) [22]	*Amino Acids*	Meta-analysis	BCAA supplementation	Moderate benefit on soreness and muscle recovery	Nutritional Therapy
Wahl P et al. (2017) [23]	*J Strength Cond Res*	RCT	Aqua cycling	No added benefit over passive recovery	Exercise Recovery Modality
Schroeter S et al. (2024) [24]	*Dtsch Z Sportmed*	Review	Compression & thermotherapies	Effective when applied early, for both prevention and treatment	Thermal & Mechanical

**Table 3:** Methodological Quality Assessment of Included Studies.

Author (Year)	Study Type	Quality Assessment Tool	Score/Level	Quality Rating	Notes
Kancherla A (2023) [1]	Management Update	—	Scoping summary	Moderate	Lacks systematic methodology
Sonkodi B et al. (2022) [2]	Research	—	Experimental study	Moderate	Good mechanistic insight, but no control group
Heiss R et al. (2024) [5]	Imaging Study	—	Descriptive imaging	Moderate	High technical quality; lacks comparison group
Longo V et al. (2016) [6]	Imaging Study	—	Descriptive imaging	Moderate	Ultrasound focused; no longitudinal follow-up
Hotfiel T et al. (2018) [7]	Review	AMSTAR 2	6/11	Moderate	Good background, lacks structured bias assessment
Xue X et al. (2023) [8]	RCT	PEDro	7/10	High	Good methodology, limited blinding
Wolska B et al. (2023) [9]	RCT	PEDro	6/10	Moderate	Missing dropout reporting, no power analysis
Mizumura K, Taguchi T (2024) [10]	Review	AMSTAR 2	7/11	Moderate–High	Strong theoretical background, weak search strategy
Wiecha S et al. (2024) [11]	Umbrella Review Protocol	ROBIS	Low risk of bias	High	Systematic protocol, registered and reproducible
Wilke J, Behringer M (2021) [12]	Review	AMSTAR 2	8/11	High	Includes fascia-specific pathophysiology, well-structured
Fleckenstein J et al. (2021) [13]	RCT	PEDro	7/10	High	Well-controlled autonomic analysis
Akehurst H et al. (2021) [14]	RCT	PEDro	6/10	Moderate	Limited by small sample and short follow-up
Chen R et al. (2024) [15]	Meta-analysis	ROBIS	Low risk of bias	High	Robust synthesis, adequate heterogeneity control
Hotfiel T et al. (2018) [7]	Review	AMSTAR 2	6/11	Moderate	Lacks explicit inclusion/exclusion criteria
Gussoni M et al. (2023) [16]	RCT	PEDro	5/10	Moderate	Null results; minimal reporting of participant flow
Iodice P et al. (2019) [17]	Therapy Study	PEDro (adapted)	6/10	Moderate	Applied vibration, lacks control arm
Romain C et al. (2017) [18]	Nutritional Study	PEDro	5/10	Moderate	Small sample; plausible results; well reported
Scudamore EM et al. (2021) [19]	Performance Study	PEDro (adapted)	6/10	Moderate	Functional outcomes only; weak blinding
Farias-Junior LF et al. (2019) [20]	Comparative Study	PEDro (adapted)	5/10	Moderate	Lacks pre-specified hypothesis
Doma K et al. (2021) [21]	Acute Effects Study	PEDro	6/10	Moderate	Good experimental setup; no long-term follow-up
Weber MG et al. (2021) [22]	Meta-analysis	ROBIS	Low risk of bias	High	Well-conducted with clear inclusion/exclusion criteria
Wahl P et al. (2017) [23]	RCT	PEDro	6/10	Moderate	Well conducted; no superiority found
Schroeter S et al. (2024) [24]	Review	AMSTAR 2	7/11	High	Good methodological structure, solid synthesis

Note: Despite the absence of a pre-specified hypothesis, the study showed acceptable methodological quality and outcome reporting consistent with PEDro criteria for moderate rating.

## Data Availability

No new data were created or analyzed in this study. Data sharing is not applicable to this article.
